# The Role of microRNA-133 in Hemocyte Proliferation and Innate Immunity of *Scylla paramamosain*

**DOI:** 10.3389/fimmu.2021.812717

**Published:** 2022-01-27

**Authors:** Yunfei Zhang, Yongyong Lai, Xiujuan Zhou, Fei Zhu

**Affiliations:** Key Laboratory of Applied Technology on Green-Eco-Healthy Animal Husbandry of Zhejiang Province, College of Animal Science and Technology, College of Veterinary Medicine, Zhejiang Agriculture and Forestry University, Hangzhou, China

**Keywords:** microRNA-133, hemocyte proliferation, innate immunity, *Scylla paramamosain*, white spot syndrome virus, *Vibrio parahaemolyticus*

## Abstract

MicroRNAs (miRNAs) are important signaling regulators that are involved in regulating the innate immunity of crustacean. However, few studies focus on the role of crustacean miRNAs in the cellular immunity have been reported. In this study, we showed that the expression of miR-133 was significantly up-regulated in the mud crab *Scylla paramamosain* after infection by white spot syndrome virus (WSSV) or *Vibrio parahaemolyticus*. The anti-miRNA oligonucleotide AMO-miR-133 was used to knock down miR-133 expression in *S. paramamosain*. The number of WSSV copies increased significantly in WSSV-infected crabs after miR-133 knockdown. Knockdown of miR-133 also enhanced the mortality rates of WSSV-infected and *V. parahaemolyticus-*infected mud crabs, and it significantly enhanced the expression of the astakine, which was confirmed by real-time quantitative PCR and western blot analysis. The data also indicate that miR-133 may affect hemocyte proliferation in *S. paramamosain* by regulating *astaki*ne expression. miR-133 Knockdown enhanced the apoptosis or phagocytosis of crab hemocytes, and increased the mortality of mud crabs after WSSV or *V. parahaemolyticus* infection. These results indicate that miR-133 is involved in the host immune response to WSSV and *V. parahaemolyticus* infection in mud crabs. Taken together, our research provides new insights for the control of viral or vibrio diseases in *S. paramamosain.*

## Introduction

MicroRNAs (miRNAs) are highly conserved endogenous small non-coding RNAs, which regulate gene expression at post-transcriptional levels and protein synthesis in eukaryotes (1). miRNAs play major roles in regulating biological processes, including cell proliferation, cell differentiation, apoptosis, DNA replication, and signal transduction (2–4). In crustaceans, miRNAs have been reported to be involved in the immune response to pathogen infections. For example, the expression level of miR-146 and miR-132 were significantly up-regulated in *Vibrio parahaemolyticus-*infected mud crabs (*Scylla paramamosain*) (5). The expression level of some miRNAs changed in *V. parahaemolyticus*-infected shrimp (*Litopenaeus vannamei*), including 47 miRNAs that were up-regulated and 36 miRNAs that were down-regulated (6). Similarly, Zhu et al. identified more than 50 kinds of miRNAs that responded to *Vibrio alginolyticus* infection in *Marsupenaeus japonicus*, and the expression levels of some of them were up-regulated (7). These reports indicate that crustacean miRNAs are associated with the process of *Vibrio* infection and may play an important role in regulation of innate immunity.

Furthermore, some researchers reported that the overexpression of miR-7 and miR-217 in the Chinese mitten crab (*Eriocheir sinensis*) was beneficial to white spot syndrome virus (WSSV) infection (8, 9). In contrast, some microRNAs in the Chinese white shrimp (*Fenneropenaeus chinensis*) were found to participate in the host response to challenge with WSSV. In another study, researchers found that the expression of 10 miRNAs in *F. chinensis* was up-regulated post-WSSV infection (10). Thus, miRNAs may also play a major role in the crustacean response to viral infection.

As one of the most important mud crab species in southeastern China, *S. paramamosain* has high economic value (11). Currently, the crab breeding industry in China is undergoing vigorous development and the mud crab aquaculture production exceeded 161 thousand tons in 2019 (12). WSSV and *V. parahaemolyticus* are two common pathogens that can infect mud crabs. However, to date, an effective method to prevent and treat WSSV and *V. parahaemolyticus* infection does not exists. Some reports have shown that hemocytes play an important role in cellular immune defense, and they can secrete several immune-related cytokines that participate in the humoral immune response (13–15). The miR-133 was first cloned and identified from mammalian muscle tissue in 2003 (16). Several studies have shown that miR-133 can significantly affect the proliferation of skeletal muscle and regeneration of myocardium in mice (17, 18), and its overexpression can significantly inhibit the proliferation of mouse vascular smooth muscle cells (17). Biological studies have confirmed that miR-133 overexpression can inhibit the proliferation of cancer cells (19, 20). Moreover, miR-133 is widely involved in the proliferation and differentiation of cardiomyocytes (21). And miR-133 can decrease cardiomyocyte apoptosis by mediating the expression of apoptosis-related genes in the hearts of mice (22).

In summary, miR-133 plays a positive role in regulating cell proliferation and apoptosis. The miR-133 was found to be up-regulated in hemocytes of *S. paramamosain* when response to WSSV and *V. parahaemolyticus* infection (23, 24). Thus, we propose that miR-133 may have similar biological functions in *S. paramamosain.* In addition, Non-specific immunity played a vital role in the defense of crustaceans against viruses, among which cellular immune response is an important part, especially because they rely on innate immunity to defend against viral infection (25). WSSV and *V. parahaemolyticus* are the main pathogens of mud crabs, and they cause great losses to the aquaculture industry. In this study, we evaluated the role of miR-133 on astakine (a hematopoietic cytokine in crustaceans) expression and cell proliferation and its effects on innate immunity and microbe pathogen infection in *S. paramamosain* **Table 1**.

**Table 1:** Universal and specific primers used in this study.

Primer Name	Primer Seqeunce (5′ to 3′)	Purpose
GAPDH-F	ACCTCACCAACTCCAACAC	for GAPDH expression
GAPDH-R	CATTCACAGCCACAACCT	
β-actin-F	ACCACTGCCGCCTCATCCTC	for β-actin expression
β-actin-R	CGGAACCTCTCGTTGCCAATGG	
STAT-F	GACTTCACTAACTTCAGCCTCG	for STAT expression
STAT-R	GAGCTGAGTCTGTCTTAATGTTATCC	
Astakine-F	CACCAGGTAGTAATCAGGGA	for Astakine expression
Astakine-R	AAGGCACCCAACTTCTCA	
MCM7-F	ACTTTGCTAACGCCAATCCAC	for MCM7 expression
MCM7-R	CTACGCTGTCATCGACGAACC	
proPO-F	ATGAAAGAGGAGTGGAGATG	For proPO expression
proPO-R	GTGATGGATGAGGAGGTG	
Myosin-F	GCCGAGATAAGTGTAGAGGAA	For Myosin expression
Myosin-R	AGTGGGGTTCTGTCCAAG	
Toll-like receptor-F	TGTTGCCAGAGCAGAAGGT	forToll-like receptor expression
Toll-like receptor-R	TTCCGTGAATGAACGAAGG	
Relish-F	CAGGTACACCTTTGTGACCGT	for Relish expression
Relish-R	CCTTCTACTTAGGGCATTTCG	
CAP-F	GCCTTTACCAACGGCTTCTTC	for Crustin antimicrobial peptide expression
CAP-R	ACAGTAGCTTCCATGCAATTC	
WSSV-F	TATTGTCTCTCCTGACGTAC	for WSSV expression
WSSV-R	CACATTCTTCACGAGTCTAC	
AMO-miR-133	AGCTGGTTGAAGGGGACC	for miR-133 knockdown
AMO-miR-133 scrambled	CTAGGAGTGGAAGGCGTC	
U6-F	CGCTTCGGCAGCACATATAC	for U6 expression
U6-R	TTCACGAATTTGCGTGTCAT	
RT-miR-133	GTCGTATCCAGTGCAGGGTCCGAGGTATTCGCACTGGATACGACACAGCT	for reversing miR-133
miR-133-F	CGTTGGTCCCCTTCAACC	for miR-133 expression
miR-133-R	AGTGCAGGGTCCGAGGTATT	

## Methods And Materials

### Preparation of Crabs and Pathogens

Healthy *S. paramamosain* (average weight 90-95 g, average carapace width 8.0 cm and carapace length 5.8 cm) were purchased from a market in Hangzhou, China, and acclimated in the aerated seawater at 25°C for 3-4 days before being used in the experiments. Three crabs were randomly selected to detect by a mentioned method described (26, 27) to ensure that the crabs were healthy before the experiments. WSSV used in this study was extracted from tissues of infected crayfish (*Procambarus clarkii*) following a previously described method (26). *V. parahaemolyticus* (ATCC 17802) was stored in an ultra-low temperature freezer in the laboratory and cultured in 2216E medium at 28°C. *V. parahaemolyticus* was collected in 1 ml phosphate buffered saline (PBS), washed three times with PBS, and used to infect the crabs following a previously described method (27).

### Silencing of *S. paramamosain* miR-133, WSSV or *V. parahaemolyticus* Challenge, and Survival Analysis

The sequence of *S. paramamosain* mir-133 was (5’-GCCCUGCUUUAGCUGGCUGAAUCCGGGCCAAAUUGUUAUUCAUAGAGCAGCAUUUGGUCCCCUUCAACCAGCUGUAGUUGGCAUUCUGAGCAAC-3’). The sequence of miR-133 was (5’-TTGGTCCCCTTCAACCAGCTGT-3’). To knockdown crab miR-133 expression, the anti-miRNA-133 oligonucleotide (AMO-miR-133: 5’-AGCTGGTTGAAGGGGACC-3’) was injected into the crab at a dose of 0.5 nmol/crab. As a control, the sequence of AMO-miR-133 was scrambled, and shown as AMO-miR-133-scramble. The experiment divided into two parts: immune index detection and survival analysis. For the first part, experimental mud crabs were distributed randomly into seven groups (3 crabs/group), with three replicates per group. Five groups were treated by intramuscular injection of 100 μL of PBS (control), AMO-miR-133 (0.5 nmol/crab), AMO-miR-133-scramble (0.5 nmol/crab), WSSV (10^6^ copies/mL), or *V. parahaemolyticus* (10^5^ CFU/mL), respectively. The other two groups were injected with a 100 μL mixture of AMO-miR-133 (0.5 nmol/crab) and either (WSSV (10^6^ copies/mL) or *V. parahaemolyticus* (10^5^ CFU/mL), respectively. At 12 h after the first injection, the anti-miRNA-133 oligonucleotide was injected into each crab.

The experimental grouping for survival analysis was the same as that described above, except AMO-miR-133-scramble group. Each group consisted of 10 crabs, and they were injected with the same doses as described above. Water in each group was replaced with fresh seawater every 24 h, at which time we recorded the number of dead crabs.

### RNA Extraction and cDNA Synthesis

The miRNA was extracted from healthy, WSSV-infected or *V. parahaemolyticus-*infected mud crabs hemocytes. And the miRNA of AMO-miR-133-scramble-treated or AMO-miR-133-treated *S. paramamosain* was also extracted from hemocytes. The extractions were performed following the manufacturer’s instructions for the miPure Cell/Tissue miRNA kit (Vazyme, Jiangsu, China). The collected miRNA samples were stored at -70°C prior to use. The miRNA first strand cDNA synthesis kit (Vazyme) was used to synthesize cDNA according to the manufacturer’s instruction. The PCR program was 5 min at 25°C, 15 min at 50°C, and 5 min at 85°C.

The total RNA from healthy or AMO-miR-133-treated *S. paramamosain* hemocytes was extracted using Trizol reagent according to the manufacturer’s instructions, and cDNA was synthesized using the PrimeScript RT reagent kit (TaKaRa, Dalian, China).

### Real-Time Quantitative PCR (RT-qPCR)

To monitor gene expression changes in *S. paramamosain* treated with AMO-miR-133 at 24 h post-treatment, three samples of *S. paramamosain* hemocytes were collected for each treatment and the control. The total RNA was extracted from the collected samples and cDNA was synthesized. Samples were analyzed using RT-qPCR following previously described methods (28). The PCR program was 30 s at 95°C; 45 cycles of 5 s at 95°C, 30 s at 60°C; 60 s at 95°C; 30 s at 55°C, and 30 s at 95°C. GAPDH was used as the internal control (28–30). The primers used for RT-qPCR were listed in **Supplementary Table 1**. The data for expression of each gene were evaluated using the 2^–ΔΔct^ method (28).

### WSSV Copy Analysis

Five healthy *S. paramamosain* from the WSSV group were injected with WSSV (10^6^ copies/mL) diluted in 100 μL of PBS, and five mud crabs injected with 100 μL of PBS served as the control group. *S. paramamosain* in the WSSV + AMO-miR-133 group were injected with a 100 μL mixture of AMO-miR-133 (0.5 nmol/crab) and WSSV (10^6^ copies/mL). Twelve hours after the first injection, the anti-miRNA-133 oligonucleotide was injected into the crabs. Hemocyte samples from three individuals from each group were collected at different times post-injection (0, 24, 48, 72 h) and we used absolute RT-qPCR to monitor WSSV copy numbers in *S. paramamosain* hemocytes.

The program was as follows: 1 cycle for 4 min at 50°C, followed by 45 cycles of 45 s at 95°C, 45 s at 52°C, and 45 s at 72°C. We collected hemocytes from *S. paramamosain* at different times post-challenge. Hemocyte DNA was extracted using a DNA isolation kit (Generay, Shanghai, China) following the manufacturer’s instructions. According to a previously described method (31), the viral load in 200 ng samples of *S. paramamosain* hemocyte DNA was then calculated. We used VP28 protein specific primers (5’-TTGGTTTCAGCCCGA-GATT-3’ and 5’-CCTTGGTCAGCCCCTTGA-3’) and TaqMan fluorescent probe (5’-FAM-TGCTGCCGTCTCCAA-TAMRA-3’) to measure the number of WSSV genomic copies.

### Superoxide Dismutase (SOD), Phenoloxidase (PO), and Catalase (CAT) Activities and Total Hemocyte (THC) Counts

SOD and PO activities were measured following a previously described method (31), as was serum CAT activity (26). The THC counts were assayed using a Burker hemocytometer (Erma, Tokyo, Japan). Measurements were made following a previously described method (32).

### Cell Culture, AMO-miR-133 Transfection, and Reactive Oxygen Species (ROS) Analysis

*S. paramamosain* hemocytes were collected and cultured following a previously described method (28). They were cultured at 28°C for 24 h, and then Lipofectamine 2000 (Invitrogen, USA) was used to transfect AMO-miR-133 (5 pmol/each well) into the cells. The PBS group served as the control. The cells were cultured in 96-well plates for different amounts of time (24, 36, and 48 h), and hemocyte proliferation levels were measured at each time point (28).

For ROS analysis, hemocytes were collected and cultured as described above. At 24 h after transfection, we measured the generated ROS in the hemocytes of the AMO-miR-133 group and the control group following a previously described method (33). We used a fluorescence labeling instrument (BioTek, Winooski, VT, USA) to measure the wavelengths of excitation at 488 nm and emission at 525 nm. The generation of ROS in cells was observed under an inverted fluorescence microscope (Nikon, Tokyo, Japan).

### Phagocyte Rate Counting by Flow Cytometry

*V. parahaemolyticus* was cultured at 37°C for 12 h on a constant temperature shaker, followed by harvesting using centrifuge at 4000 rpm for 10 min at 4°C and resuspension in PBS. Samples were placed in a water bath at 80°C for 60 min and then incubated with 5 mg/mL fluorescent isothiocyanate (FITC; Sigma, USA) at 25°C for 90 min. PBS was used to wash labelled *V. parahaemolyticus* until the supernatant was clear. WSSV samples were labelled following a previously described method (34).

The hemocyte samples were collected from each group and placed in pre-cooled ACD-B (pH 4.6; citric acid 0.96 g/200 mL; sodium citrate 2.64 g/200 mL; glucose 2.94 g/200 mL; NaCl 0.24 g/200 mL) at a ratio of 1:1. The samples were centrifuged at 300 g x 5 min at 4°C to collect hemolymph cells.

### Apoptosis Analysis of Crab Hemocytes

At 24 h after injection with AMO-miR-133, the pathogen, or their mixture, hemocytes were collected from each group, and the apoptosis analysis was performed following a previous method (34). Cell apoptosis of each sample was examined by flow cytometry, and the cells in quadrant 4 with low propidium iodide (PI) and high annexin V staining was considered to be apoptotic.

### Western Blot

The hemocyte samples from each group were mixed with anticoagulant solution at a 1:1 ratio and centrifuged at 800 g at 4°C for 5 min to collect hemocytes. Cell lysis buffer for western blot analysis and IP were used to lyse hemocytes. After full lysis, the samples were centrifuged at 10,000–14,000 g at 4°C for 10 min, and the supernatants were collected. Protein supernatants were separated through 12% SDS-PAGE and then transferred to nitrocellulose filter membranes. The subsequent western blot procedure followed a previously described method (35). Rabbit anti-astakine antiserum and anti-GAPDH were purchased from HuaAn Biotechnology Co. (Hangzhou, China). Horseradish peroxidase-conjugated goat anti-rabbit secondary antibody was purchased from Bioker Biotechnology Company (Hangzhou, China).

### Statistical Analysis

Relative gene expression was calculated using the 2^−△△CT^ method. SPSS 19.0 software (IBM, Armonk, NY, USA) was used to analyze data. Statistical significance was determined using the Duncan multiple comparison test or one-way analysis of variance for two group comparisons. All data are presented as the mean ± standard deviation of three independent experiments. In all cases, *P* < 0.05 was considered to be statistically significant.

## Results

### miR-133 Expression Following *V. parahaemolyticus* or WSSV Challenge

**Figure 1:**
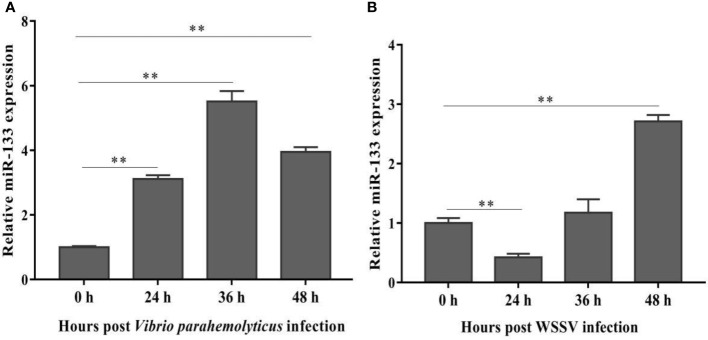
*S. paramamosain* miR-133 expression following *V. parahaemolyticus* **(A)** (24 h *P* = 0.0000080, 36 h *P* = 0.000019, 48 h *P* = 0.0000053) or WSSV infection **(B)** (24 h *P* = 0.00064, 36 h *P* = 0.29, 48 h *P* = 0.0048) as measured using RT-qPCR. Hemocytes were collected at different times (0, 24, 36, 48 h) post challenge, and the miRNA of healthy, WSSV-infected or *V. parahaemolyticus*-infected mud crabs was extracted from hemocytes. Mud crab *GAPDH* was used as the control. Data presented were the mean ± standard deviation of three independent experiments. Significant difference between different samples is indicated by **P < 0.01.

To determine whether miR-133 is involved in the response of *S. paramamosain* to WSSV or *V. parahaemolyticus* infection, hemocytes of healthy, WSSV-infected, and *V. parahaemolyticus-*infected mud crabs were collected to measure the expression of miR-133. The expression of miR-133 in *S. paramamosain* was obviously up-regulated after *V. parahaemolyticus* infection (**Figure 1A**), and a similar result was detected after WSSV infection, especially at 36 and 48 h (**Figure 1B**). These results suggest that miR-133 has potential as a new strategy for treating and preventing WSSV and *V. parahaemolyticus* infections. However, the expression of miR-133 was reduced by WSSV infection at 24 h, and we speculate that WSSV had not completely invaded the cells at this time point.

### Effect of miR-133 Knockdown on Expression of miR-133 and Immune-Related Genes

**Figure 2:**
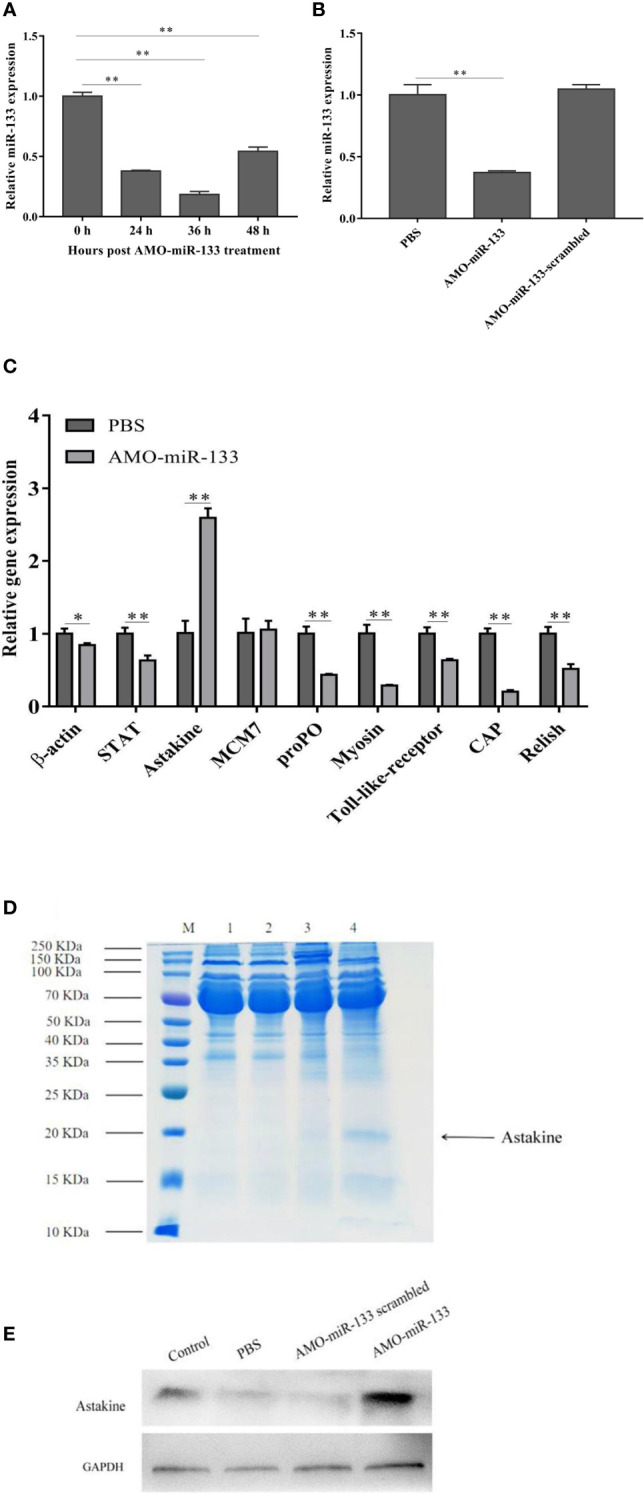
Knockdown of miR-133. The miRNA of healthy, AMO-miR-133-scramble-treated or AMO-miR-133-treated *S. paramamosain* was extracted from hemocytes. **(A, B)** Expression levels of miR-133 in hemocytes of *S. paramamosain* detected by RT-qPCR, **(A)** (24 h *P* =0.000028, 36 h *P* = 0.000014, 48 h *P* = 0.00020), **(B)** AMO-miR-133 *P* = 0.000028, AMO-miR-133 scrambled *P* = 0.26. **(C)** Relative expression of nine immune-related genes (*β-actin (P = 0.021), STAT (P = 0.0011), astakine (P = 0.00021), MCM7 (P = 0.75), proPO (P = 0.00053), myosin (P = 0.00050), Toll-like receptor (P = 0.00050), CAP (P = 0.000057), Relish (P = 0.0017)*) after AMO-miR-133 treatment. **(D)** Mud crabs were treated for 24 h with the indicated concentration of AMO-miR-133, then the cell lysate was prepared for SDS-PAGE (M: Marker; 1: Control; 2: PBS; 3: AMO-miR-133 scrambled; 4: AMO-miR-133) or **(E)** western blot analysis. Mud crab GAPDH was used as the control. Data presented were the mean ± standard deviation of three independent experiments. Significant difference between different samples is indicated by *P < 0.05 and **P < 0.01.

The expression of miR-133 in hemocytes from 24 to 48 h post-AMO-miR-133 treatment was inhibited significantly (**Figure 2A**). The expression of miR-133 in hemocytes did not differ significantly between the PBS group and the AMO-miR-133-scrambled group at 24 h (**Figure 2B**).

We evaluated the immune pathways by analyzing the expression of nine important genes (*β-actin, STAT, astakine, MCM7, proPO, myosin, Toll-like receptor, CAP*, and *Relish*) after samples were treated with AMO-miR-133 for 24 h. **Figure 2C** shows the expression of immune-related genes in *S. paramamosain* at 24 h post-AMO-miR-133 treatment. The expression level of astakine increased significantly compared with that of the PBS group, and this finding was verified by SDS-PAGE (**Figure 2D**) and western blot analysis (**Figure 2E**). These results suggest that miR-133 might be involved in regulating the expression of astakine.

### Effect of miR-133 Knockdown on CAT Activity

**Figure 3:**
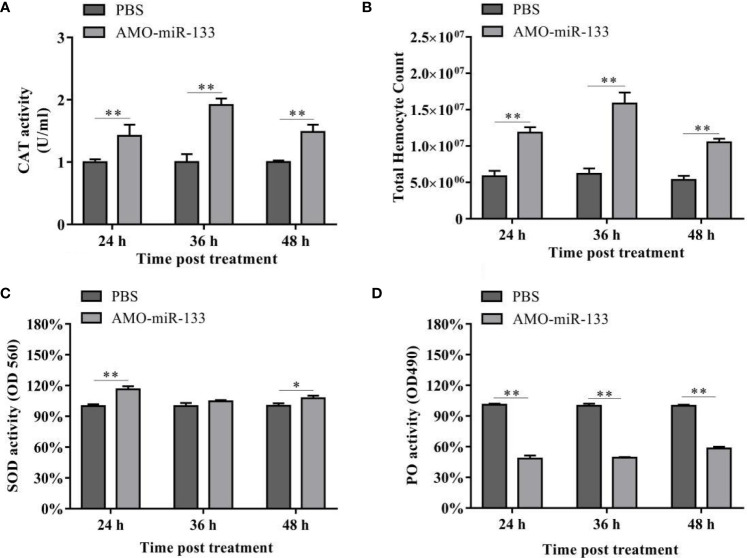
**(A)** Effect of AMO-miR-133 on CAT activity of healthy *S. paramamosain* at 24, 36, and 48 h after AMO-miR-133 treatment. **(B)** Effect of AMO-miR-133 on the total hemocyte counts at 24, 36, and 48 h after AMO-miR-133 treatment. **(C)** Effect of AMO-miR-133 on SOD activity at 24, 36, and 48 h after AMO-miR-133 treatment. **(D)** Effect of AMO-miR-133 on PO activity at 24, 36, and 48 h after AMO-miR-133 treatment. The results are shown as mean ± standard deviation of three independent experiments. Significant difference between different samples based on t-test analysis is indicated by *P < 0.05 and **P < 0.01.

**Figure 4:**
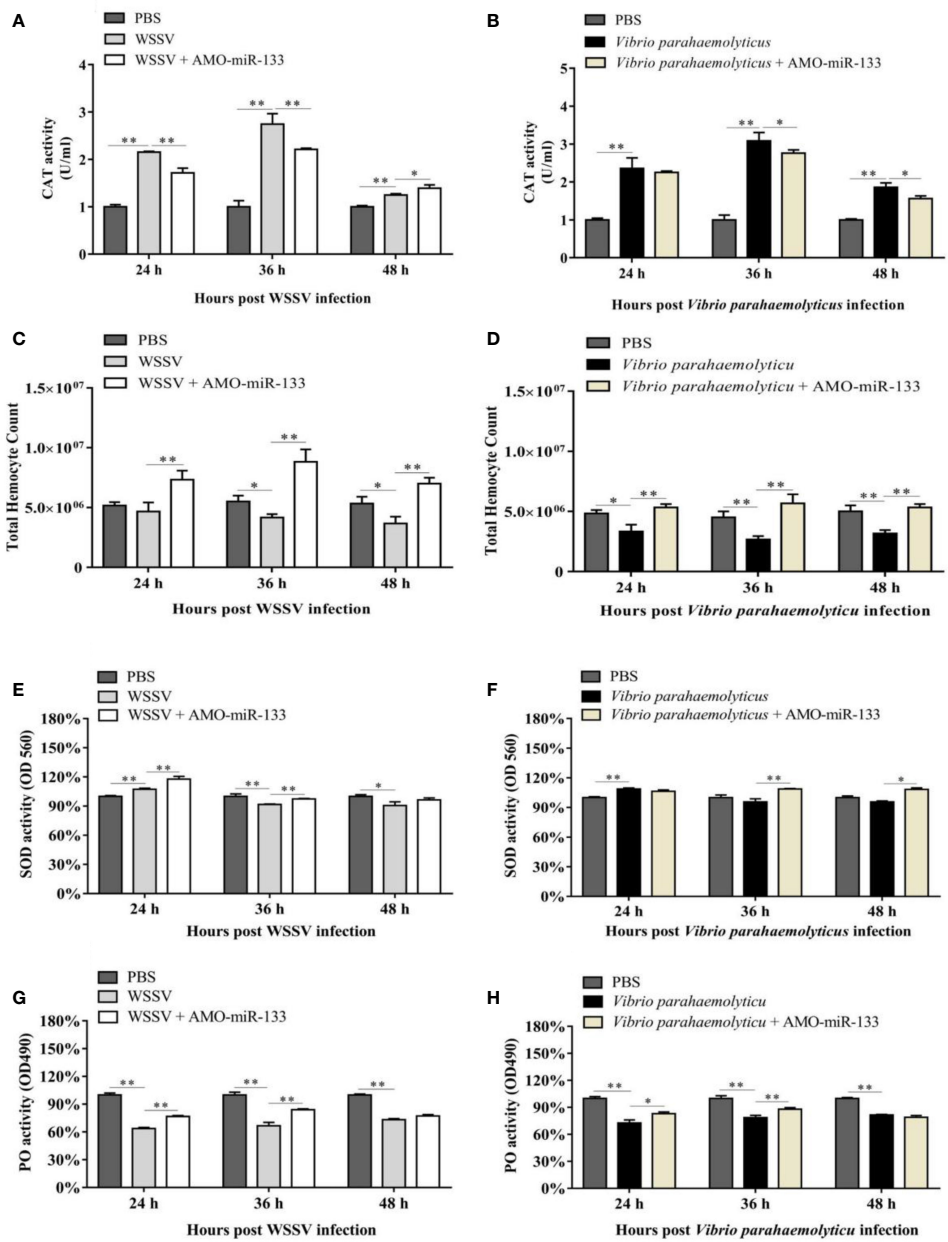
Samples were taken at different times (24, 36, and 48 h) post-treatment. **(A)** CAT activity in the WSSV (24 h *P* = 0.0000023, 36 h *P* = 0.000046 and 48 h *P* = 0.00035) or WSSV + AMO-miR-133 group (24 h *P* = 0.0017, 36 h *P* = 0.00062 and 48 h *P* = 0.033). **(B)** CAT activity in the *V. parahaemolyticus* (24* h P* = 0.0012, 36 h *P* = 0.000065 and 48 h *P* = 0.00025) or *V. parahaemolyticus* + AMO-miR-133 group (24 h *P* = 0.55, 36 h *P* = 0.031 and 48 h *P* = 0.020). **(C)** Effect of AMO-miR-133 on THC of WSSV-challenged mud crabs. WSSV group (24 h *P* = 0.35, 36 h *P* = 0.016 and 48 h *P* = 0.024). WSSV + AMO-miR-133 group (24 h *P* = 0.0031, 36 h *P* = 0.0017 and 48 h *P* = 0.001641289). **(D)** The effect of AMO-miR-133 on THC of *V. parahaemolyticus-*challenged mud crabs. *V. parahaemolyticus* group (24 h *P* = 0.016, 36 h *P* = 0.0053 and 48 h *P* = 0.0053). *V. parahaemolyticus* + AMO-miR-133 group (24 h *P* = 0.0058, 36 h *P* = 0.0031 and 48 h *P* = 0.00078). **(E)** SOD activity in the WSSV (24 h *P* = 0.0013, 36 h *P* = 0.0046 and 48 h *P* = 0.021) or WSSV + AMO-miR-133 group (24 h *P* = 0.0043, 36 h *P* = 0.000052 and 48 h *P* = 0.079). **(F)** SOD activity in the *V. parahaemolyticus* (24 h *P* = 0.00086, 36 h *P* = 0.14 and 48 h *P* = 0.055) or *V. parahaemolyticus* + AMO-miR-133 groups (24 h *P* = 0.078, 36 h *P* = 0.0022 and 48 h *P* = 0.018). **(G)** PO activity after WSSV or WSSV + AMO-miR-133 challenge. WSSV group (24 h *P* = 0.0000096, 36 h *P* = 0.00029 and 48 h *P* = 0.0023). WSSV + AMO-miR-133 group (24 h *P* = 0.00031, 36 h *P* = 0.0023 and 48 h *P* = 0.15). **(H)** PO activity after *V. parahaemolyticus* or *V. parahaemolyticus* + AMO-miR-133 treatment. *V. parahaemolyticus* group (24 h *P* = 0.00029, 36 h *P* = 0.00067 and 48 h *P* = 0.000021). *V. parahaemolyticus* + AMO-miR-133 group (24 h *P* = 0.010, 36 h *P* = 0.0080 and 48 h *P* = 0.069). The results are shown as mean ± standard deviation of three independent experiments. Significant difference between different samples is indicated by *P < 0.05 and **P < 0.01.

To evaluate the impact of miR-133 on CAT activity, we measured the CAT activity in hemocytes of *S. paramamosain*. After AMO-miR-133 treatment, the activities of CAT were significantly enhanced at different times (24, 36, and 48 h) compared with the PBS group **(** **Figure 3A** **)**. The CAT activity of the WSSV + AMO-miR-133 group was higher than that of the WSSV group at 48 h (**Figure 4A** **)**. In contrast, the CAT activity of the *V. parahaemolyticus* + AMO-miR-133 group was reduced compared with that of the *V. parahaemolyticus* group at 36 and 48 h (**Figure 4B**).

### Effect of miR-133 Knockdown on the THC

In the AMO-miR-133 group, the THC increased significantly from 24 to 48 h (**Figure 3B**), but the value at 48 h was significantly lower than that at 36 h. In the WSSV group, the THC decreased from 24 to 48 h (**Figure 4C**). However, in the WSSV + AMO-miR-133 group, the THC was significantly higher than that of the PBS and WSSV groups. *V. parahaemolyticus* challenge also showed the similar results (**Figure 4D**). These data indicate that miR-133 may affect hemocyte proliferation in *S. paramamosain*.

### Effect of miR-133 Knockdown on SOD Activity

Significant differences in SOD activities were observed among the three groups at different times (24, 36 and 48 h) post-AMO-miR-133 treatment. For the AMO-miR-133 group, the SOD activity of crabs was significantly higher than that of the PBS group, especially at 24 h (**Figure 3C**). For the WSSV and *V. parahaemolyticus* groups, SOD activities were significantly enhanced at 24 h compared with the PBS group (**Figure 4E**). However, at 36 and 48 h, the SOD activity decreased in the *V. parahaemolyticus* group. In the WSSV group, the SOD activity was decreased (**Figure 4F**). The SOD activity of the WSSV + AMO-miR-133 group was higher than that of the WSSV group at 36 and 48 h post-challenge (**Figure 4E**). The *V. parahaemolyticus* challenge produced a similar result (**Figure 4F**).

### Effect of miR-133 Knockdown on PO Activity

Compared to the PBS group, the PO activities of *S. paramamosain* treated with AMO-miR-133 were significantly lower from 24 to 48 h (**Figure 3D**). After WSSV or *V. parahaemolyticus* infection, the PO activities of the two groups decreased significantly compared with that of the PBS group (**Figures 4G, H** **)**. The PO activities of the AMO-miR-133 +WSSV and AMO-miR-133 + *V. parahaemolyticus* groups were higher than those of the WSSV or *V. parahaemolyticus* groups, respectively, from 24 to 36 h. However, the PO activities at 48 h were significantly lower than those at.

### Effect of miR-133 Knockdown on ROS Activity and Hemocyte Proliferation

**Figure 5:**
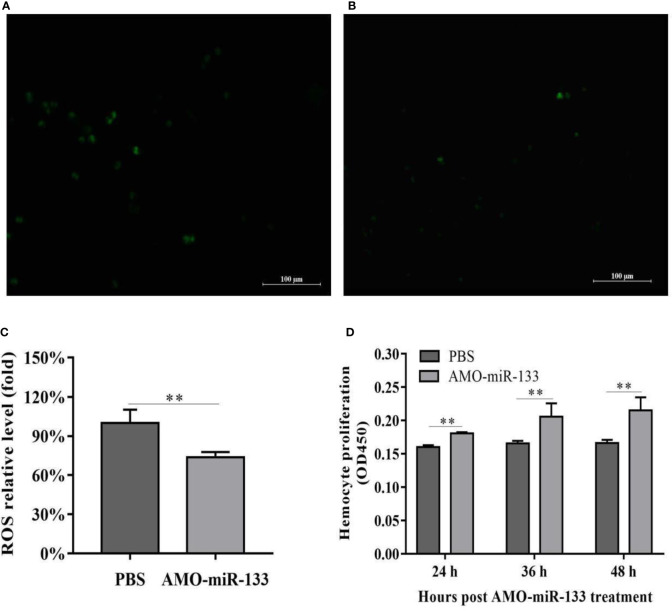
ROS activity and proliferation of hemocytes. **(A)** PBS group. **(B)** AMO-miR-133 group. **(C)** ROS activity columnar statistical chart. **(D)** Hemocyte proliferation columnar statistical chart. Bars represent the means of three individual means ± SD. Significant difference between different samples is indicated by **P < 0.01.

To evaluate the effect of miR-133 on hemocyte proliferation and ROS activity, we cultured *S. paramamosain* hemocytes in 96-well transparent plates and subsequently treated them with AMO-miR-133 or PBS. The ROS activity of the AMO-miR-133 group was significantly lower than that of the PBS group **(** **Figures 5A–C** **)**, which echoed the results of SOD activity. The levels of hemocyte proliferation in the AMO-miR-133 group was higher than that of the PBS group at different times (24, 36, and 48 h) post-treatment **(** **Figure 5D** **)**.

### Effect of miR-133 Knockdown on Apoptosis

**Figure 6:**
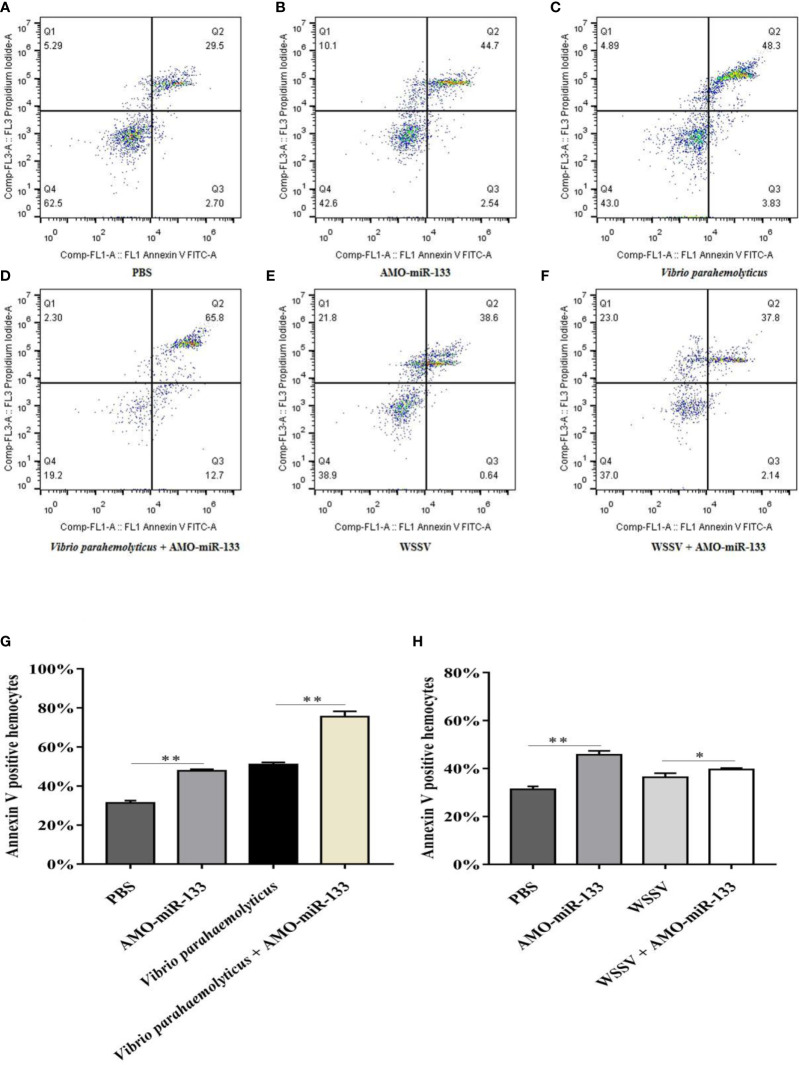
Hemocyte apoptosis assay. **(A)** PBS group. **(B)** AMO-miR-133group. **(C)** *V.o parahaemolyticus* group. **(D)** *V. parahaemolyticus* + AMO-miR-133 group. **(E)** WSSV group. **(F)** WSSV + AMO-miR-133 group. **(G)** Bar graph of apoptosis after challenge with *V. parahaemolyticus*. **(H)** Statistical bar graph of apoptosis caused by WSSV infection. Significant difference between different samples is indicated by *P < 0.05 and **P < 0.01.

To assess how miR-133 affects hemocyte apoptosis, we measured the apoptosis rates in the AMO-miR-133 group. The hemocyte apoptosis rate was greatly up-regulated in the AMO-miR-133 group compared with the PBS group (**Figures 6A, B** **)**. In response to WSSV or *V. parahaemolyticus* challenge, the apoptosis rates at 24 h were significantly higher than that of the PBS group (**Figures 6C, E** **)**. After *V. parahaemolyticus* challenge, apoptosis rates were significantly enhanced in the *V. parahaemolyticus +* AMO-miR-133 group relative to the PBS group (**Figure 6D**), and similar results were obtained after WSSV challenge (**Figure 6F**). These results show that knockdown of miR-133 expression increased hemocyte apoptosis compared with that of healthy *S. paramamosain* and WSSV-infected (**Figure 6H**) or *V. parahaemolyticus-*infected crabs (**Figure 6G**).

### Effect of miR-133 Knockdown on Hemocyte Phagocytosis

**Figure 7:**
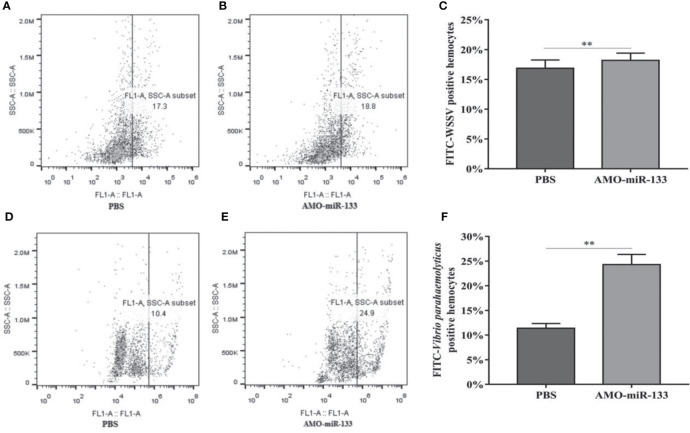
Flow cytometry assay of hemocyte phagocytosis. Inactivated WSSV was labeled with FITC. **(A)** PBS group. **(B)** AMO-miR-133 group. **(C)** Bar graph of phagocytosis of WSSV. **(D)** PBS group. **(E)** AMO-miR-133 group. **(F)** Bar graph of phagocytosis of *V. parahaemolyticus.* Significant difference between different samples is indicated by **P < 0.01.

To determine how miR-133 affects the phagocytosis of hemocytes, we investigated hemocyte phagocytosis in *S. paramamosain* at 24 h post-AMO-miR-133 treatment. The hemocyte phagocytic activity against WSSV and *V. parahaemolyticus* was enhanced compared with that of the PBS group (**Figure 7**).

### Effect of miR-133 Knockdown on Virus Infection

**Figure 8:**
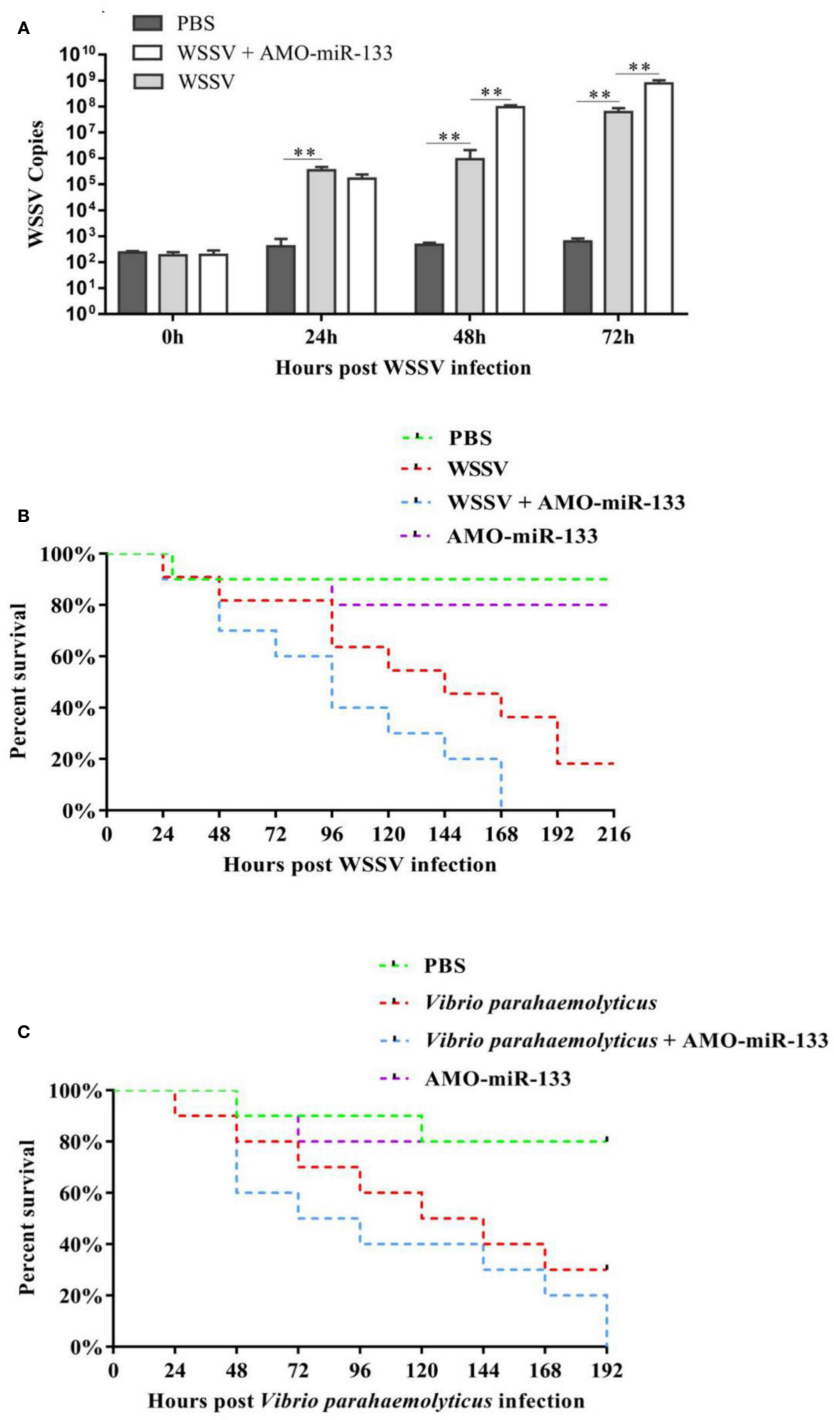
**(A)** To analyze WSSV copies after WSSV challenge, hemocytes were collected at different times (0, 24, 48, 72 h) post-WSSV infection and assessed using RT-qPCR. **(B)** Cumulative mortality of WSSV- or **(C)** *V. parahaemolyticus*-challenged *S. paramamosain* treated with AMO-miR-133. Each group contained 10 individuals. Significant difference between different samples is indicated by **P < 0.01.

To determine the function of miR-133 during WSSV infection, the virus copy numbers in *S. paramamosain* from the PBS group, WSSV group, and WSSV + AMO-miR-133 group were measured at different time after challenge (**Figure 8A**). At 24 h post-challenge, the virus copy number in the WSSV + AMO-miR-133 group was slightly lower than that of the WSSV group, but the difference was not statistically significant. From 48 to 72 h, the virus copy number in the WSSV + AMO-miR-133 group significantly increased relative to that of the WSSV group.

### Effect of miR-133 Knockdown onSurvival Rate

**Figure 8B** showed the cumulative survival rate of *S. paramamosain* from the PBS group, WSSV group, and WSSV + AMO-miR-133 group. A significantly higher mortality rate was found in the WSSV + AMO-miR-133 group compared with the WSSV group at 168 h post-infection. We obtained similar results after *V. parahaemolyticus* infection (**Figure 8C**). However, AMO-miR-133 itself had no adverse effects on *S. paramamosain*. These results suggest that miR-133 knockdown reduced the survival rate of WSSV-infected *S. paramamosain* or *V. parahaemolyticus*-infected *S. paramamosain*.

## Discussion

Although miRNAs have been implicated in the regulation of cell proliferation and signal transduction (3, 4) and are involved in numerous biological processes, their molecular functions are still largely unknown. Moreover, few studies of the roles of miRNAs in crustaceans have been conducted. Among crustaceans, miRNAs were first studied in *Daphnia pulex*, which is often used as a model animal (36). Crustaceans rely on innate immunity to protect themselves from invading microbes (37). For example, the hard carapace is the first line of defense for crabs, and it provides an effective physical barrier (38). If the hard carapace fails to resist pathogens, innate immunity, which consists of humoral and cellular responses, is stimulated (39).

Antisense oligonucleotides (AMOs) have been used to silence specific miRNAs *in vivo* in order to investigate the function of miRNAs in crustaceans (40, 41). Recent reports have shown that some miRNAs may play a major role in inhibiting virus invasion in mammals, and knockdown of miRNAs can increase the sensitivity of hosts to viral infection (42, 43). A variety of evidence has shown that miRNAs play an important role in the interaction between virus and host. Host miRNA can affect the replication and transmission of virus in the host by targeting virus or host genes, and a single miRNA often has multiple targeted genes (43).

To date, the study of miR-133 in the process of viral infection of hosts has mostly focused on mammals. Compared with vertebrates, few studies of the roles of miRNAs in the invertebrate response to virus infection have been reported (41). Mammal miR-133 was initially isolated from muscle cells and was later found to be involved in different physiological processes (44). For a long time, miR-133 was regarded as a muscle-specific miRNA, which may participate in myogenic diseases and regulate myoblast differentiation (45, 46). *In vitro*, miR-133 was shown to inhibit cardiac hypertrophy (47), and in a variety of cancers, it was reported to suppress cell proliferation, apoptosis, and migration (17, 48, 49).

The mud crab *S. paramamosain* has become an economically important aquaculture species in southern and eastern China (50), but it is negatively impacted by diseases. Because miRNAs may have a potential antiviral role in viral infection, crustacean miRNAs need to be studied. A previous study performed using RT-qPCR technology to detect the change of miRNA expression in mud crabs infected with WSSV revealed that miR-133 was up-regulated in response to WSSV infection at 2 and 12 h post-infection (23). Thus, we choose miR-133 for further study.

The expression of mud crab miR-133 was significantly higher in hemocytes of WSSV-challenged crabs than in healthy crabs. This result suggests that mud crab miR-133 may participate in innate immunity and defend against WSSV or *V. parahaemolyticus* infection. To evaluate the potential function of miR-133 in the innate immune system of mud crabs, we examined the effect of AMO-miR-133 on immune-related gene expression, the survival of WSSV- or *V. parahaemolyticus*-challenged crabs, and other immune-related parameters. Several immune-related genes related to pattern recognition receptors and signaling pathways have been reported (51–55). For example, the JAK/STAT signaling pathway was found to be important for vertebrate and invertebrate defense against viral infection (55, 56). The STAT gene, which is a major component of the JAK/STAT pathway, was identified in *Penaeus monodon*, *F. chinensis* and *S. paramamosain* (57, 58). Astakine is an important hematopoietic cytokine that is directly involved in hematopoiesis, and it plays a major role in proliferation and differentiation of hematopoietic stem cells (59, 60). In a previous study, we found that crab MCM7 may contribute to the immune response to WSSV or *V. alginolyticus* infection by regulating phagocytosis, apoptosis, and other immune parameters (61). Myosin was also shown to be involved in antiviral defense and cell skeleton construction in shrimp (62). Additionally, cationic antimicrobial peptide (CAP) in crabs can trigger host innate immunity against microbial invasion (63), and the antimicrobial peptide transcription factor Relish can regulate apoptosis in WSSV-infected *S. paramamosain* (52).

In this study, we used AMO-miR-133 to inhibit the expression of miR-133 in *S. paramamosain.* The expression levels of innate immune factors *β-actin*, *STAT*, *proPO*, *myosin*, *Toll-like receptor*, *CAP*, and *Relish* were significantly down-regulated after the knockdown of miR-133 in *S. paramamosain*, whereas that of *astakine* was up-regulated significantly. These results indicate that miR-133 may participate in immune regulation of mud crabs. Additionally, several immune parameters were detected in AMO-miR-133-treated *S. paramamosain*, which may help us understand the immune function of miR-133. THC increased significantly in the AMO-miR-133 group, and the increase was also found in the *in vitro* hemocyte proliferation experiments. This increase may be related to the up-regulation of *astakine* expression, as astakine was shown to induce a strong hematopoiesis response in crayfish (59). When we explored the function of *astakine* in mud crabs, we found that its expression was highest in hemocytes. After interfering with its expression, THC and cell proliferation were significantly reduced, which indicated that astakine affected hemocyte proliferation in the crabs (28).

High ROS levels lead to immune dysfunction, which is detrimental to aerobic organisms, and enzymatic and non-enzymatic antioxidants are used to scavenge redundant ROS (64, 65). Hydrogen peroxide can be catalyzed effectively by CAT, which helps maintain balance of its generation and efficient elimination (66). In our study, CAT and SOD activities were enhanced in the AMO-miR-133 group and in pathogen-challenged crabs, which suggests that knockdown of miR-133 improved the antioxidant capacity of the crabs. The ROS results confirmed this conclusion.

PO is important for the host immune response to several pathogens (67). We found that PO activity was down-regulated in AMO-miR-133-treated crabs, which suggests that miR-133 may participate in the immune response to WSSV or *V. parahaemolyticus* infection by regulating PO activity. The improved immune functional parameters in AMO-miR-133-treated crabs may be related to the up-regulation of astakine. We detected higher hemocyte apoptosis in the WSSV and *V. parahaemolyticus* groups than in the PBS group, and the WSSV + AMO-miR-133 and *V. parahaemolyticus* + AMO-miR-133 groups had higher hemocyte apoptosis than the WSSV or *V. parahaemolyticus* groups. Similarly, knocking down miR-133 improved hemocyte phagocytic activity in the immune response to WSSV or *V. parahaemolyticus* infection. These data suggest that miR-133 may participate in the immune response to WSSV or *V. parahaemolyticus* infection by affecting hemocyte apoptosis and phagocytosis. Similar findings were reported previously for MCM7 and Relish in *S. paramamosain* (52, 61). The higher number of virus copies and greater mortality observed in the WSSV + AMO-miR-133 group indicated that miR-133 may be involved in inhibiting WSSV replication. We also recorded higher mortality in the *V. parahaemolyticus* + AMO-miR-133 group during *V. parahaemolyticus* challenge.

In conclusion, our data suggest that miR-133 plays an important role in the innate immunity of *S. paramamosain.* miR-133 Knockdown enhanced the apoptosis or phagocytosis of crab hemocytes and increased the mortality of mud crabs after WSSV or *V. parahaemolyticus* infection. Based on the results, miR-133 most likely affects innate immunity of mud crabs especially in hemocyte proliferation by regulating *astakine* expression. When mud crabs are infected with WSSV or *V. parahaemolyticus*, miR-133 may change *astakine* expression to cope with pathogen infection. During this process, some important immune parameters, such as ROS, hemocyte apoptosis, and CAT and PO activities are affected by miR-133.

## Data Availability Statement

The original contributions presented in the study are included in the article/**Supplementary Material**. Further inquiries can be directed to the corresponding author.

## Author Contributions

We have made substantial contributions to the conception or design of the work; or the acquisition, analysis, or interpretation of data for the work. FZ and YZ conceived and designed research. YZ, YL, and XZ conducted experiments. FZ and YZ analyzed data. FZ and YZ wrote the manuscript. The corresponding author is responsible for ensuring that the descriptions are accurate and agreed by all authors. We have drafted the work or revised it critically for important intellectual content. We have approved the final version to be published. We agree to be accountable for all aspects of the work in ensuring that questions related to the accuracy or integrity of any part of the work are appropriately investigated and resolved. YZ: Investigation, Data curation, Writing-Original draft preparation, Validation, Software. YL: Investigation, Sample preparation, Software. XZ: Investigation, Sample preparation. FZ: Conceptualization, Data curation, Writing-Reviewing and Editing. All authors contributed to the article and approved the submitted version.

## Funding

This work was financially supported by Basic Public Welfare Research Project of Zhejiang Province (LY20C190001).

## Conflict of Interest

The authors declare that the research was conducted in the absence of any commercial or financial relationships that could be construed as a potential conflict of interest.

## Publisher’s Note

All claims expressed in this article are solely those of the authors and do not necessarily represent those of their affiliated organizations, or those of the publisher, the editors and the reviewers. Any product that may be evaluated in this article, or claim that may be made by its manufacturer, is not guaranteed or endorsed by the publisher.
