# Supplementary material for: The Role of microRNA-133 in Hemocyte Proliferation and Innate Immunity of Scylla paramamosain
Source: Front Immunol. 2022 Jan 27;12:812717. doi: 10.3389/fimmu.2021.812717 (PMC8828940; doi:10.3389/fimmu.2021.812717)
Supplement: Supplementary file 1 [file Table_1.docx]

**Supplementary Table 1**

Universal and specific primers used in this study.

| Primer Name | Primer Seqeunce (5′ to 3′ ) | Purpose |
| --- | --- | --- |
| GAPDH-F | ACCTCACCAACTCCAACAC | for GAPDH expression |
| GAPDH-R | CATTCACAGCCACAACCT |  |
| β-actin-F | ACCACTGCCGCCTCATCCTC | for β-actin expression |
| β-actin-R | CGGAACCTCTCGTTGCCAATGG |  |
| STAT-F | GACTTCACTAACTTCAGCCTCG | for STAT expression |
| STAT-R | GAGCTGAGTCTGTCTTAATGTTATCC |  |
| Astakine-F | CACCAGGTAGTAATCAGGGA | for Astakine expression |
| Astakine-R | AAGGCACCCAACTTCTCA |  |
| MCM7-F | ACTTTGCTAACGCCAATCCAC | for MCM7 expression |
| MCM7-R | CTACGCTGTCATCGACGAACC |  |
| proPO-F | ATGAAAGAGGAGTGGAGATG | For proPO expression |
| proPO-R | GTGATGGATGAGGAGGTG |  |
| Myosin-F | GCCGAGATAAGTGTAGAGGAA | For Myosin expression |
| Myosin-R | AGTGGGGTTCTGTCCAAG |  |
| Toll-like receptor-F | TGTTGCCAGAGCAGAAGGT | forToll-like receptor expression |
| Toll-like receptor-R | TTCCGTGAATGAACGAAGG |  |
| Relish-F | CAGGTACACCTTTGTGACCGT | for Relish expression |
| Relish-R | CCTTCTACTTAGGGCATTTCG |  |
| CAP-F | GCCTTTACCAACGGCTTCTTC | for Crustin antimicrobial peptide expression |
| CAP-R | ACAGTAGCTTCCATGCAATTC |  |
| WSSV-F | TATTGTCTCTCCTGACGTAC | for WSSV expression |
| WSSV-R | CACATTCTTCACGAGTCTAC |  |
| AMO-miR-133 | ACAGCTGGTTGAAGGGGACCAA | for miR-133 knockdown |
| AMO-miR-133 scrambled | CTAGGAGTGGAAGGCGTC |  |
| U6-F | CGCTTCGGCAGCACATATAC | for U6 expression |
| U6-R | TTCACGAATTTGCGTGTCAT |  |
| RT-miR-133 | GTCGTATCCAGTGCAGGGTCCGAGGTATTCGCACTGGATACGACACAGCT | for reversing miR-133 |
| miR-133-F | CGTTGGTCCCCTTCAACC | for miR-133 expression |
| miR-133-R | AGTGCAGGGTCCGAGGTATT |  |
